# The role of matrix metalloproteinase expression levels in the pathogenesis of tympanosclerosis

**DOI:** 10.1007/s00405-025-09509-3

**Published:** 2025-06-04

**Authors:** Semih Van, Doğukan Özdemir, Seda Koç Şahin, Şule Öztürk, Dursun Mehmet Mehel, Ayşe Çeçen

**Affiliations:** 1 Department of Otolaryngology and Head & Neck Surgery Adıyaman Training and Research Hospital, Adıyaman, Turkey; 2 Department of Otolaryngology and Head & Neck Surgery, Samsun University Samsun Training and Research Hospital, Samsun, Turkey; 3 Department of Medical Pathology, Samsun University Samsun Training and Research Hospital, Samsun, Turkey; 4 Department of Public Health, Samsun University Samsun Training and Research Hospital, Samsun, Turkey; 5 Department of Otolaryngology and Head and Neck Surgery, Samsun Training and Research Hospital, Samsun, Turkey

**Keywords:** Tympanosclerosis, Myringosclerosis, Metalloproteinase, MMP-2, MMP-9

## Abstract

**Purpose:**

Tympanosclerosis is a disease characterized by the accumulation of hyaline calcareous deposits in the tympanic membrane, tympanic cavity, ossicular chain, ligaments, and rarely, in the mastoid. Matrix metalloproteinases, which are involved in physiological and pathologic processes, are also likely to play a role in tympanosclerosis. In our study, we aimed to investigate the presence of MMP 2 and 9 in patients with chronic otitis media with tympanosclerosis and its possible effect on tympanosclerosis severity and prognosis.

**Methods:**

In our study, 45 patients with myringosclerosis or myringosclerosis and intratympanic sclerosis who underwent endoscopic transcanal tympanoplasty due to COM (chronic otitis media) were included in the study group, and 35 patients with only COM, without tympanosclerosis, were included in the control group. Immunohistochemical staining of the mucosa samples of the eardrum and middle ear were compared with MMP-2 and MMP-9 in the study and control groups.

**Results:**

The rate of MMP-2 staining (64.3%) was higher than the control group (42.9%), and the rate of MMP-9 staining (16.7%) was lower than the control group (51.4%) (*p* < 0.05). The rate of MMP-9 staining in the middle ear mucosa samples (31.0%) was statistically lower than the control group (57.1%) (*p* < 0.05).

**Conclusion:**

In our study, high levels of MMP-2 staining in the study group and low levels of MMP-9 staining in the control group help to elucidate the complex relationship between both metalloproteinases. Compared with the inadequacy of animal models of tympanosclerosis to mimic the chronic process, our study reveals the late results of MMP-2 and MMP-9 in patients with tympanosclerosis.

## Introduction

Tympanosclerosis (TS) is a disease of the eardrum and middle ear characterized by hyaline degeneration [1–3]. This degenerative process begins in the submucosal connective tissue and spreads to all layers of connective tissue [2]. Especially in cases of tympanosclerosis, in which the eardrum and middle ear are affected together, bilateral hearing loss may develop as a result of fixation in the ossicles. Developing hearing loss is an important health problem in terms of social and treatment cost [1–4].

Considerable work has been done to establish the etiopathogenesis of tympanosclerosis. Although its morphology is well known, there is still no consensus on the etiology and pathogenesis of tympanosclerosis. As possible causes of tympanosclerosis, middle ear infections, immunologic factors, genetic predisposition, oxidative stress, and trauma are blamed. The most blamed of these factors are chronic and recurrent infections of the middle ear and ventilation tube applications [5–7].

Histologically, tympanosclerosis is characterized by fibroblast infiltration of the lamina propria, increased collagen fibers and hyalized tissue formation. Damage to the lamina propria occurs due to trauma and inflammation. The damage is tried to be repaired by increasing the collagen fibers. In the fibrotic tissue formed during this repair process, calcium and phosphate accumulation occurs as a result of changes in extracellular calcium stores. As a result, white, mucous raised tympanosclerotic plaques are formed [1, 8]. Calcium accumulation sometimes progresses to the formation of cartilage and bone tissue [9].

There is no definitive curative treatment for tympanosclerosis other than surgery. Due to the difficulties in surgical treatment and the high probability of complications in surgical treatment, it is more important to prevent its development. In addition, recurrences are frequent after surgery and hearing results are poor [10, 11].

Matrix metalloproteinases (matrixins; MMP) are a family of 30-member endopeptidases that cleave the extracellular matrix (ECM), released from epithelial and endothelial cells, fibroblasts and inflammatory cells. Their common feature is that they have zinc-containing catalytic regions. They are synthesized and released inactively, activated in the extracellular area, and each specifically breaks down one or more substrates. These proteinases play an important role in many pathologic processes such as embryogenesis, normal tissue remodeling, wound healing, angiogenesis, atheroma, arthritis, cancer, and tissue ulceration. MMPs are key mediators in the remodeling of normal ECM, which is involved in development, tissue morphogenesis, and repair. In addition, MMPs are also responsible for the unintended destruction of the ECM, which constitutes the pathophysiology of various diseases [12–15].

Matrix metalloproteinase-2 and 9 disrupt Type 4 collagen, which is the main component of basement membranes [16, 17]. MMP-2 and 9 are involved in physiologic processes that occur during membrane remodeling and repair and play a crucial role in pathologies such as rheumatoid arthritis, aortic aneurysms, myocardial infarction, septic shock, liver disease, tumor invasion, and metastasis [18, 19]. Although MMP-2 and MMP-9 have similar functions in the destruction of the ECM and tissue injuries, their timing of expression is different.

In the literature, there are no studies investigating the presence of MMP-2 and MMP-9 together in sclerotic tissues and their effect on the disease in patients with tympanosclerosis. In previous studies, MMP-2 and 9 levels in tympanosclerotic foci were evaluated separately in animal models with relatively short-term inflammation [20, 21]. In a study conducted with human serum samples, serum levels of MMP-2 and 9 were found to be significantly higher in patients with tympanosclerosis [22].

However, these high serum levels may not be sufficient to show the exact source of MMPs. Because these proteinases can be secreted by a variety of cells, including epithelial cells, fibroblasts, macrophages, and other inflammatory cells. In our study, we aimed to investigate the presence of MMP-2 and 9 in patients with chronic otitis media (COM) with tympanosclerosis and their possible effects on tympanosclerosis severity and prognosis.

## Materials and methods

Our study was conducted on 80 patients who underwent surgery for COM in the Department of Otorhinolaryngology and Head and Neck Surgery between January 2022 and March 2023 after obtaining informed consent forms from the individuals to be included in the study and approval of the ethics committee (Ethics Committee / date: 29/12/2021 / decision no: 2021/628). Patients who underwent endoscopic type 1 tympanoplasty surgery, had preoperative pure tone audiometry, and had no ear discharge for at least 3 months were included in the study. Patients who had previously undergone myringotomy, ventilation tube placement, tympanoplasty or mastoidectomy, who had clinical or radiologic findings of ear infection, cholesteatoma, granulation tissue, systemic diseases related to bone formation or destruction (such as osteopetrosis, osteogenesis imperfecta, Paget’s disease), immunodeficiency, aged younger than 18 years, and had attic perforation were excluded from the study. Patients who underwent ossicular chain reconstruction were excluded from the study. Detailed anamnesis information, physical examination findings, audiologic tests, and radiologic image reports were recorded. The patients were informed about the surgery and consent was obtained from the patients for the procedure.

Patients were divided into two groups: COM with tympanosclerosis, and COM without tympanosclerosis (control group). Forty-five patients who underwent endoscopic type 1 tympanoplasty for COM, myringosclerosis or myringosclerosis and intratympanic sclerosis were included in the study group, and 35 patients without myringosclerosis and intratympanic sclerosis were included in the control group. During tympanoplasty, samples were taken from the eardrum and middle ear mucosa in the study group, and from the eardrum perforation edges and middle ear mucosa in the control group.

All patients underwent type 1 tympanoplasty with a transcanal approach under general anesthesia. Chondroperichondrial graft material taken from the tragal region was used as a graft. Sclerotic plaques in the tympanic membrane were excised in the study group. The edges of the tympanic membrane perforation were de-epithelialized with a pick in both groups. In patients with intratympanic sclerosis, plaques around the ossicular chain were cleaned and mobilization of the system was attempted. The primary aim in the study group was to provide mobilization by cleaning the plaques affecting the mechanism and to repair the closed cavity with myringoplasty. Ossiculoplasty was planned according to audiologic results after 6 months postoperatively. Then, the perichondrial graft taken from the tragus was placed with the over-underlay technique. The perichondrial graft was supported with a whole piece of cartilage, leaving the manubrium mallei exposed. Gelfoams were placed in the middle ear to support the annulus. The tympanomeatal flap was repositioned in the external auditory canal. After observing that the edges of the perforation were completely closed with the perichondrial graft, the external auditory canal was filled with gelfoams. The incision was sutured with 4.0 rapid Vicryl. A dressing was applied and the surgery was terminated.

Pure tone audiometry tests of the patients were performed and recorded in the audiology unit of our clinic. Pure tone audiometry tests of the patients were performed at frequencies of 500, 1000, 2000 and 4000 Hz within 1 month before the surgery and at follow-ups 1 and 6 months after the surgery. Masking was applied to patients with indications. The mean air-bone conduction gap value was found by subtracting the mean bone conduction hearing threshold value from the mean airway hearing threshold value. The mean air conduction threshold (ATC), bone conduction threshold (BTC), and air-bone conduction gap (ABG) of the patients at 500, 1000, 2000, and 4000 Hz frequencies before and after the surgery were calculated and compared [23].

The tissues removed for light microscopic evaluation were fixed in buffered 10% neutral formaldehyde solution for 2 days. During macroscopic evaluation, the softness and hardness of the tissues were evaluated. The hard tissues were also subjected to decalcification in formic acid-sodium citrate solution. All tissues were subjected to routine tissue follow-up procedures and paraffin blocks were prepared. Sections of 4–6 micrometer thickness were taken with a Leitz-1512 microtome and hematoxylin and eosin (H&E) staining was performed. The prepared sections were evaluated under the light microscope (Olympus BX53) by a pathologist who was unaware of the study and control groups. Images were taken with a photomicroscope (Olympus DP72). Histopathologically, the presence and/or proliferation of fibroblasts, formation of collagen fibers, hyalinization, calcification, ossification and the presence of chondroblast-like cells were evaluated. In the evaluation, tympanosclerotic tissue samples were divided into three groups (8). Fibroblast proliferation and uniform collagen fibers were seen in type 1 tympanosclerotic tissue. In type 2 tympanosclerotic tissue, significant accumulation of hyaline collagen surrounding fibroblasts was observed. In type 3 tympanosclerotic tissue, the collagen fibers of the connective tissue were irregular, thicker, and tightly arranged. The collagen fibers were tightly and closely monitored, similar to the appearance of the bone-like matrix. Calcification, ossification, and the presence of chondroblast-like cells were evaluated as presence or absence.

From the removed tissues, 3-micrometer-thick sections were cut onto Poly-L-Lysine coated slides using a Leitz-1512 microtome from paraffin blocks. Sections to be subjected to immunohistochemistry were deparaffinized overnight in a 37-degree oven. The sections were stained with matrix metalloproteinase-2 (MMP-2) (monoclonal mouse antibody (6E3F8), catalog number: ab86607, Abcam, England) and matrix metalloproteinase-9 (MMP-9) (multiclonal recombinant rabbit antibody (RM1020), catalog number: ab283575, Abcam, England) using an automatic immunohistochemical staining machine (Ventana, Benchmark, XT, USA). Immunohistochemistry-stained preparations were evaluated under an Olympus BX53 light microscope. MMP-2 and MMP-9 assessment was performed according to the presence or absence of staining.

The SPSS (version 22.0, SPSS Inc.) package program was used for statistical analysis of the data. Conformity to normal distribution was examined using the Kolmogorov-Smirnov test. Continuous variables are given as mean ± standard deviation (minimum-maximum), and categorical variables are given as number (s) and percentage (%). Categorical variables were analyzed using Pearson’s Chi-square test or, where appropriate, Fisher’s exact test. The independent sample t-test (Student’s t-test) was used to compare two independent groups that fit the normal distribution, and a t-test (paired t- test) was used to compare two dependent groups. Statistically, *p* < 0.05 was considered significant.

## Results

Of the 80 patients included between January 2022 and March 2023, two were excluded from the study due to graft failure and one due to cholesteatoma. The study was evaluated on the data of a total of 77 cases, 35 of which were controls (45.45%) and 42 (54.54%) were studies. Of these, 34 (44.2%) were male and 43 (55.8%) were female. The mean age of the study group was 36.45 ± 13.87 years, and the mean age of the control group was 38.40 ± 12.48 years. There was no statistically significant difference between the study and control groups in terms of sex and age (*p* = 0.47, *p* > 0.52).

There was no statistically significant difference between the study and control groups in terms of the proportions of perforation sizes (*p* = 0.33).

Of the sclerotic plaque specimens in the study group, 15 (35.7%) were soft and 27 (64.3%) were hard type. Sclerotic plaque samples taken from the eardrum in the study group were divided into types by H&E staining under light microscopy. Of the sclerotic tissues, type 1 was observed in four (9.5%), type 2 in 12 (28.5%), and type 3 in 26 (61.9%) tympanosclerosis. The proportion of plaques with type 3 tympanosclerosis in the study group (74.1%) was statistically significantly higher than the proportion of soft plaques (33.3%) (*p* = 0.027).

The relationship between the degree of rigidity of sclerotic plaques in the study group and the immunohistochemical staining status of MMP-2 and MMP-9 was examined. Nineteen (70.4%) of the hard plates were stained with MMP-2, and eight (29.6%) of the soft plates were stained with MMP-2. There was no statistically significant difference between hard and soft plates in terms of MMP-2 staining (*p* = 0.270). Four (57.1%) of the hard plates were stained with MMP-9, and three (42.9%) of the soft plates were stained with MMP-9. There was no statistically significant difference between hard and soft plates in terms of MMP-9 staining status (*p* = 0.686).

Although the rate of calcification in the study group (16.7%) was higher than in the control group (5.7%) in the eardrum samples of the study and control groups, there was no statistically significant difference between the groups (*p* = 0.170). Although the rate of ossification in the study group (16.7%) was higher in the control group (3.6%), there was no statistically significant difference between the groups (*p* = 0.065). The proportion of chondroblast-like cells in the study group (81.0%) was lower than in the control group (100.0%). A statistically significant correlation was found between the study and control groups in terms of the presence of chondroblast-like cells (*p* = 0.007).

The eardrum samples of the study and control groups were compared according to their immunohistochemical staining with MMP-2 and MMP-9. The rate of MMP-2 staining in the study group (64.3%) was found to be higher than in the control group (42.9%). A statistically significant relationship was found between the MMP-2 staining rates of the study and control groups (*p* = 0.049). The MMP-9 staining rates in the study group (16.7%) were found to be lower than the control group (51.4%) and a statistically significant relationship was found between them (*p* = 0.001) (Table 1).

**Table 1:** Immunohistochemical evaluation of MMP-2 and MMP-9 in intergroup eardrum samples

	Groups		Test statistics	
	Study n:42	Control n:35	Test value	*p-value*
**Eardrum samples**, *n*(%*)				
MMP-2			χ2 = 3.856	**0.049**
(-)	15 (35.7%)	20 (57.1%)		
(+)	27 (64.3%)	15 (42.9%)		
MMP-9			χ2 = 10.52	**0.001**
(-)	35 (83.3%)	17 (48.6%)		
(+)	7 (16.7%)	18 (51.4%)		

n: number, *: Column percentage, χ2: Pearson Chi-square Test

The middle ear mucosa samples of the study and control groups were compared according to their immunohistochemical staining with MMP-2 and MMP-9. The rate of MMP-2 staining in the study group (54.8%) was higher than in the control group (54.3%), and there was no statistically significant difference between them (*p* = 0.96). The rate of MMP-9 staining in the study group (31.0%) was lower than in the control group (57.1%), which was statistically significant (*p* = 0.021) (Table 2).

**Table 2:** Immunohistochemical evaluation of MMP-2 and MMP-9 in intergroup middle ear mucosal samples

	Groups		Test statistics	
	Study n:42	Control n:35	Test value	*p-value*
**Specimens of middle ear mucosa**, *n*(%*)				
MMP-2			χ2 = 0.002	0.967
(-)	19 (45.2%)	16 (45.7%)		
(+)	23 (54.8%)	19 (54.3%)		
MMP-9			χ2 = 5.347	**0.021**
(-)	29 (69.0%)	15 (42.9%)		
(+)	13 (31.0%)	20 (57.1%)		

n: number, *: Column percentage, χ2: Pearson Chi-square Test

The mean airway threshold values ​​of the study and control groups were compared. When the mean airway threshold values ​​measured at the 6th month after surgery were examined, the mean of the study group was 29.26 ± 13.23, which was found to be statistically significantly higher than the control group (18.91 ± 6.20) (*p* < 0.001) (Table 3).

**Table 3:** Comparison of mean ACT values between the groups

	Groups		Test statistics	
	Study group	Control group	Test value	*p-value*
Preoperative Mean ACT (*DB*) $\:\stackrel{-}{\varvec{x}}$±SD	39.21 ± 12.95	36.20 ± 9.00	t = 1.162	0.249
Postoperative 1st month Mean ACT (*DB*) $\:\stackrel{-}{\varvec{x}}$±SD	29.69 ± 12.60	21.49 ± 7.12	t = 3.419	**0.001**
Postoperative 6th month Mean ACT (*DB*) $\:\stackrel{-}{\varvec{x}}$±SD	29.26 ± 13.23	18.91 ± 6.20	t = 4.248	**< 0.001**

dB: Decibel, ATC avg: Airway threshold mean, $\:\stackrel{-}{x}$: Mean, *SD*: Standard deviation, *t*: Independent Sample T-Test

The mean air-bone gap values of the study and control groups were compared. When the mean values measured at the 6th postoperative month were examined, the mean value of the study group was 13.43 ± 7.47, which was found to be statistically significantly higher than that of the control group (5.23 ± 3.44) (*p* < 0.001) (Table 4).

**Table 4:** Comparison of mean values of ABG between groups

	Groups		Test statistics	
	Study group	Control group	Test value	*p-value*
Preoperative Mean ABG (*dB*) $\:\stackrel{-}{\varvec{x}}$±SD	22.40 ± 9.47	21.77 ± 6.94	*t* = 0.329	0.743
Postoperative 1st month Mean ABG (*dB*) $\:\stackrel{-}{\varvec{x}}$±SD	13.48 ± 8.56	8.71 ± 5.42	*t* = 2.960	**0.006**
Postoperative 6th month Mean ABG( *dB*) $\:\stackrel{-}{\varvec{x}}$±SD	13.43 ± 7.47	5.23 ± 3.44	*t* = 6.344	**< 0.001**

dB: Decibel, ABG avg: Mean air-bone conduction gap, $\:\stackrel{-}{x}$: Mean, *SD*: Standard deviation, *t*: Independent Sample T-Test

In our study, preoperative and postoperative airway threshold, bone conduction threshold, and air-bone conduction gap mean values were compared in both groups. In the control group, unlike the study group, a statistically significant difference was observed in the mean values of airway and air-bone gap between the 1st postoperative month and the 6th postoperative month.

The relationship between the mean values ​​of ACT, BTC, and ABG according to the MMP-2 and MMP-9 immunohistochemical staining status of sclerotic plaques in the study group was examined. No significant difference was found in all mean values ​​in those stained with MMP-2 compared with those not stained with MMP-2. The mean values ​​of postoperative 6th month airway threshold in the study group were found as 23.14 ± 5.66 in those stained with MMP-9 and were statistically significantly lower than those not stained with MMP-9 (30.49 ± 14.01) (*p* = 0.031). The mean value of preoperative air-bone gap in the study group was found as (15.42 ± 8.42) in those stained with MMP-9 and were statistically significantly higher than those not stained with MMP-9 (*p* = 0.031).

## Discussion

Although its morphology is well known, the etiology of tympanosclerosis is still not fully understood. Immunologic reactions, genetic predisposition, infection, trauma, and free oxygen radicals are possible underlying causes of tympanosclerosis [5]. In our study, the fact that all patients in both groups were diagnosed as having chronic otitis media and had no history of ear surgery including myringotomy and tube placement allows us to evaluate the development of tympanosclerosis after chronic inflammation.

Similarly, Akyıldız classified tympanosclerotic plaques according to their surgical detachment characteristics. Soft plaques are defined as plaques that are soft by palpation and can be easily removed. The hard type, on the other hand, is hard, white, and can hardly be removed from the surrounding tissues [1]. In our study, 15 (35.7%) of the sclerotic plaques taken from the eardrum of the study group were soft and 27 (64.3%) were hard. In the study conducted by Selçuk et al., which included 17 patients with tympanosclerosis diagnosed with COM, 23.5% of the sclerotic plaques were found to be soft, 35.4% were moderately hard, and 41.1% were hard. In the same study, tympanosclerotic plaques were examined histopathologically, otomicrically, and under light microscopy, and in the light of the histopathologic findings obtained, the specimens were classified as types I, II and III sclerotic tissues [8]. In our study, type 1 tympanosclerosis was observed in four (9.5%), type 2 in 12 (28.5%), and type 3 in 26 (61.9%) plaques on microscopic examination after H&E staining. Type 3 Tympanosclerosis was statistically significantly higher than the rate of hard plaques (74.1%) compared with soft plaques (33.3%). Also, in our study, 19 (70.4%) of the hard plates were stained with MMP-2, and eight (29.6%) of the soft plates were stained with MMP-2. Four (57.1%) of the hard plates were stained with MMP-9, and three (42.9%) of the soft plates were stained with MMP-9. There was no statistically significant difference between the groups in terms of MMP-2 and MMP-9 staining status. These data show that tympanosclerotic plaques have different histopathologic stages while completing a developmental process. As noted in a past study, hard or Type 3 plaques can be seen late in tympanosclerosis, and soft or Type 1 plaques can be seen in the early period [8]. In patients with type 1 plaques, new plaques can be seen as the process may continue even if surgical removal is performed. In type 3 plaques, the tympanosclerosis process may be completed. Recognition of these morphologic and microscopic features suggests that it may be beneficial for preoperative planning. High rates of MMP-2 staining in hard plaques compared with soft plaques suggest that MMP-2 represents the late period when the disease is more severe, and MMP-9 is less stained in both soft and hard plaques, suggesting that MMP-9 is suppressed in the late stage of tympanosclerosis. However, the lack of statistical significance between the groups can be explained by the insufficient number of patients included in the study.

Studies on MMPs have shown that early inhibition of MMP-9 activity after acute myocardial infarction helps resolve inflammation [24–27]. Kato et al. investigated the role of MMP-2 in hepatic ischemia-reperfusion injury in animal models [28]. Livers of rats with MMP-2 deficiency showed intense leukocyte infiltration and high levels of proinflammatory cytokines, and a significant percentage died within 48 h after the procedure. A high degree of increase in active MMP-9 was observed in leukocytes migrating into the liver. In addition, the DNA repair enzyme poly-ADP-ribose polymerase (PARP), previously identified as an MMP-2 substrate, showed increased activity in damaged livers with MMP-2 deficiency [29]. Researchers concluded that MMP-2 was initially protective and that its absence led to hepatic cytotoxicity and animal death due to MMP-9-mediated leukocyte infiltration as well as PARP activation. MMPs have been considered therapeutic targets for years for ulcerative colitis, Crohn’s disease, chronic obstructive pulmonary diseases such as emphysema, atherosclerosis, and arthritis [30].

It is now clear that MMPs have much broader roles in such chronic inflammatory diseases. While one stage of inflammation provides resolution of disease, the other stage leads to severe remodeling and fibrosis. Although MMP-2 and MMP-9 have similar functions in the degradation of the ECM and show overlapping expression during tissue injury, studies have shown that the timing of expression is very close but different [16, 31]. Although the presence of MMPs in disease in chronic diseases is well known, one of the challenges of understanding what they actually do in vivo is that the right animal model has not been found. Although there are many animal models of chronic diseases, none have been completely successful in summarizing the complexity of the disease. To mimic intestinal diseases, dextran sulfate sodium (DSS) is often used as a damaging agent, but if it is not given in multiple cycles, an acute inflammation is induced. MMP-9 is expressed by both infiltrated leukocytes and colonic epithelium following exposure to DSS, and symptoms associated with acute DSS resolve in rats lacking MMP-9 [32–36].

In our study, eardrum samples were stained statistically significantly higher with MMP-2 in the study group and MMP-9 in the control group. In middle ear mucosal samples, MMP-9 stained at a statistically significantly higher rate in the control group. In the study group, the high rate of staining of eardrum samples with MMP-2 was found to be consistent with the studies in the literature [16, 31]. Although middle ear mucosa samples were stained with MMP-2 at a higher rate in the study group, this result was not statistically significant. The fact that the samples taken from the middle ear mucosa did not show macroscopically sclerotic properties may have affected this result. Our study differs from previous animal studies in that the low rate of staining of eardrum and middle ear mucosa samples with MMP-9 in tympanosclerosis [20, 21].

Animal studies may be insufficient to mimic the development of tympanosclerosis in terms of duration and mechanism of occurrence. Our study reveals the long process of tympanosclerosis on the background of chronic inflammation. Gibb stated that 10–30 years were needed for the formation of clinical tympanosclerosis [37]. In the study conducted by Guo et al., MMP-9 level tended to decrease after the 4th week, suggesting that MMP-9 expression decreased in late-stage tympanosclerosis. In our study, high staining of MMP-2 and low staining of MMP-9 in the study group helps to elucidate the complex relationship between both MMPs. It was thought that the presence of high MMP-2 may suppress MMP-9 or high MMP-9 may suppress MMP-2.

In the study conducted by Ozcan et al., topical application of the MMP inhibitor doxycycline was shown to inhibit the development of experimental TS in TM and middle ear mucosa [38]. In the tympanosclerosis animal model, in which the possible role of MMP-9 together with TGF-β1 was investigated, it was reported that TGF-β1 expression increased with the development of tympanosclerosis at 8 weeks, and MMP-9 expression increased slowly from 1 to 4 weeks and decreased at 6 weeks. These results showed that the degree of expression of TGF-β1 was positively correlated with the duration of tympanosclerosis and had a bidirectional effect on MMP-9 in the 6-week period [20]. The activity of MMP-2 and MMP-9 was evaluated by immunohistochemical staining in tissues 6 weeks after the intervention in an animal model by Kurnaz et al., in which resveratrol was used to prevent tympanosclerosis. Although there was no significant difference in MMP-2 staining between the groups, a statistically significant difference was observed in MMP-9 staining of tympanic membranes between the resveratrol-treated group and the control group. In the control group eardrum, MMP-9 stained more strongly [21]. Koslowski et al. reported that MMP-2 activity reached its maximum on day 3 during the acute inflammation phase, and that activity decreased during chronic inflammation. However, MMP-2 increased again at the beginning of the fibrotic phase [31]. Goussev et al. showed that the active form of MMP-9 was elevated 1 day after spinal cord injury and returned to normal after 14 days. The active form of MMP-2 appeared on day 7 and remained elevated for at least 21 days [16]. In our study, the staining rates of MMP-2 and 9 in the study and control groups support the literature in terms of different expression timings, although they show similar functions in the breakdown of the ECM. According to the results of our study, it was thought that MMP-2 may play a role in the late and more sclerotic period of tympanosclerosis, and MMP-9 may play a role in the pre-tympanosclerosis period of COM. More long-term studies are needed to investigate the relationship between MMP-9 expression levels, which have been shown to be present in animal models of tympanosclerosis, in terms of the development of tympanosclerosis or disease recurrence on the basis of COM.

In the study conducted by Aslan et al., patients with chronic otitis media with and without tympanosclerosis were discussed, MMP-2 and MMP-9 levels in serum were measured more than healthy people, and it was concluded that enzymes that inhibit them could be used in treatment [22]. However, these high serum levels may not be sufficient to show the exact source of MMPs because these proteinases can be secreted by a variety of cells, including epithelial cells, fibroblasts, macrophages, and other inflammatory cells. Our study provides direct evidence that both MMP-2 and MMP-9 are produced locally during tympanosclerosis and/or chronic otitis media.

In the study group, a significant decrease was observed in the mean values ​​of airway and air-bone gap between preoperative and postoperative 1st month and preoperative and postoperative 6th month. In the control group, a significant decrease was observed in the mean values ​​of airway and air-bone gap between preoperative and postoperative 1st month, preoperative and postoperative 6th month, and postoperative 1st month and postoperative 6th month. The fact that no significant decrease was observed between postoperative 1st and 6th months in the study group and the control group may have been due to the negative effect of tympanosclerosis on healing and the surgical method applied (cleaning and releasing of plaques).

In our study, the relationship between MMP-2 and 9 immunohistochemical staining of sclerotic plaques and the mean values ​​of airway, bone conduction, and air-bone conduction gap was examined in the study group. In the study group stained with MMP-9, all hearing averages were found to be lower in the preoperative, and postoperative 1st and 6th months compared with those not stained. In the study group stained with MMP-9, the preoperative air-bone conduction gap and postoperative 6th month airway mean values ​​were found to be significantly lower. No relationship was found between MMP-2 staining in sclerotic plaques and hearing results. The small number of patients with widespread tympanosclerosis in the study group may have caused this significant difference not to be observed. The fact that the preoperative air-bone conduction gap and postoperative 6th month airway mean values ​​were lower in the study groups stained with MMP-9 may indicate that MMP-9 is effective in the period when tympanosclerosis is not early or more widespread. According to these results, the presence of MMP-9 staining seems to have a positive effect on hearing test results. However, the 6-month follow-up period may be insufficient to investigate the predisposition of MMP-9 to tympanosclerosis. In addition, our study constitutes a unique example in the literature in terms of investigating the relationship between MMP-2 and 9 and hearing test results in tympanosclerosis.

## Conclusion and recommendations

MMP-2 and 9, which have been studied in animal models of tympanosclerosis, were investigated in our study in the sclerotic tissues of patients with tympanosclerosis and their relationship with hearing outcomes was evaluated. Our study provides direct evidence that both MMP-2 and MMP-9 are produced locally during the process of tympanosclerosis and/or chronic otitis media.

In our study, the increased MMP-2 and decreased MMP-9 staining rates in tympanosclerosis suggest that it represents the late stage of the disease and that the atrophied mucosa is no longer able to respond to further microbial invasion. The increased MMP-9 staining rate in the control group suggests that the active immune response due to mucositis and the chronic inflammation process continuing in the COM period before tympanosclerosis increased MMP-9 expression. In addition, the lower preoperative air-bone gap and postoperative 6th month airway averages in the MMP-9 stained study group compared with those not stained suggest that MMP-9 is present during the period when tympanosclerosis is not early or widespread and positively affects postoperative recovery. The relationship between high expression levels of MMP-9 and the severity of chronic middle ear infections and the tendency to tympanosclerosis should be investigated. In addition, longer follow-up studies are needed to establish a relationship between MMP-2 and 9 staining and disease recurrence.

In conclusion, the relationship between MMP-2 and MMP-9 may play an important role in the pathogenesis of the development of tympanosclerosis against the background of chronic inflammation. However, more long-term studies are required to investigate the specific molecular mechanisms between MMP-2 and MMP-9 and determine the clinical utility of specific protease inhibitors in tympanosclerosis and other pathologic processes.
